# Dental Survey of Institutionalized Children with Autistic Disorder

**DOI:** 10.5005/jp-journals-10005-1130

**Published:** 2012-02-24

**Authors:** Gagandeep Mohinderpal Chadha, Pradnya Kakodkar, Vishwas Chaugule, Vidya Nimbalkar

**Affiliations:** Department of Community Dentistry, Dr DY Patil Dental College Pimpri, Maharashtra, India; Ex-Reader, Department of Community Dentistry, Dr DY Patil Dental College, Pimpri, Maharashtra, India; Head, Department of Pediatric Dentistry, Dr DY Patil Dental College Pimpri, Maharashtra, India; Ex-Lecturer, Department of Community Dentistry, Dr DY Patil Dental College, Pimpri, Maharashtra, India

**Keywords:** Autism, Dietary pattern, Oral hygiene practices, Oral health status, Dental needs, Institutionalized, India

## Abstract

The objective of this study was to assess the oral hygiene practices, dietary pattern, dental caries status and needs of institutionalized autistic children. The sample consisted of 35 children (28 males and 7 females) in the age group of 5 to 10 years from two institutions in Maharashtra, India. The parents of the children were interviewed regarding oral hygiene practices of their respective ward and instructed to maintain a 4-day diet chart for their children. A clinical examination was conducted using WHO dentition status and treatment needs index and a simplified oral hygiene index for ages 4 to 6 years and 7 to 10 years (deciduous and mixed dentition) was used to assess the oral hygiene. The results of diet chart analysis according to Nizel AE and Papas AS score showed the ‘at meal’ sugar exposure close to nil, while the ‘in between’ meal sugar exposure was observed to be more than three times per day among maximum children. The oral hygiene status was poor with abundance of soft debris and fair calculus accumulation. The mean caries experience (deft) in these children was 6.4. The present study provided baseline data which has been used for planning a comprehensive oral health care program.

**How to cite this article:** Chadha GM, Kakodkar P, Chaugule V, Nimbalkar V. Dental Survey of Institutionalized Children with Autistic Disorder. Int J Clin Pediatr Dent 2012;5(1):29-32.

## INTRODUCTION

Autism is defined by the Autism Society of America (ASA)^1^as: A complex developmental disability that typically appears during the first 3 years of life and is the result of a neurological disorder that affects the normal functioning of the brain, impacting development in the areas of social interaction and communication skills. It has no cure.^2^ Autism is highly heritable, although the genetics of autism is complex. The worldwide incidence of autism is about 0.2%^3^and the oral health of these children has been found to be poor for the reasons listed below:

1. Autistic children have poor self-help skills.
2. They have less learning abilities than healthy individuals.^4,5^
3. Sensory problems, such as sensitivity to the bristles of brush and to taste of toothpaste or powder, are marked.
4. Two-thirds of autistic children are hyperactive and hence have manifestations of nutritional deficiencies.
5. Autistic children have strong affinity for sweets.^6,7^
6. Atypical eating behavior occurs in about three quarters of children.
7. Autism varies widely in symptoms and severity and some people have coexisting disabilities, such as mental retardation or epilepsy thus they are most challenging dental patients.

In face of such data, there has been an increasing interest to assess the oral health status and study the dietary behavior of autistic children so that an early preventive intervention can help to avoid further occurrence of new lesions, reduce present disease status and maintain good oral health.

Thus keeping in mind, the above factors, the present study was conducted with the aim to assess the dietary pattern, oral hygiene practices, dental caries status and needs of institutionalized autistic children (Maharashtra, India) and to utilize this data for planning a comprehensive oral health care program.

## MATERIALS AND METHODS

Out of the seven institutions in Maharashtra for autistic children, our study was conducted in two such institutions namely Prasanna Autistic Centre, Prizm limited, Pune, and Ashadeep Special School, Kupwad, Miraj. The sample of our study consisted of 40 autistic children (32 males and 8 females) in the age group of 5 to 10 years. Informed consent was obtained from the parents of these children and the institution authorities. Those children whose parents did not give consent and who were uncooperative during examination were excluded from the study.

The data was collected in three parts. First, all the parents were interviewed and instructed to record the socio- demographic variables and oral hygiene practices followed by their child. Oral hygiene practices, like brushing habits, agents used, interdental aids and use of any other aids, were recorded. Next, the diet charts of the children were obtained, by asking the parents to fill a diet chart for their child for 4 days (2 days of the weekend and any 2 working days) as per the instructions given by Nizel AE and Papas AS.^8^ Later all the children were clinically examined at their institution by a single operator using sterile mouth mirror and explorer. Autistic characteristics, like hypersensitivity, lack of interactive skills, hyperactivity and uncooperative behavior, make oral examination and treatment a challenge. These hindrances during the examination were overcome; first by establishing a rapport with the child taking assistance from the institution staff in a safe environment with minimum distractions, and then using visual aids and simple and direct instructions.

Dental caries status and needs were assessed by using WHO dentition status and treatment needs index,^9^ while the oral hygiene was assessed by using a simplified oral hygiene index for ages 4 to 6 years and 7 to 10 years (deciduous and mixed dentition).^10^ For the ages 4 to 6 years, labial surfaces of the 54, 61, 82 and the lingual surface of 75 were selected. For the mixed dentition, the labial surfaces of the 54, 61, 82, 26 and the lingual surface of 75, 46 were selected.

The dietary chart for sugar consumption was analyzed for ‘at meal’ and ‘between meal’ sugar exposures as per the instructions described by Nizel AE and Papas AS.^8^ Using the sweet evaluation chart given by Nizel AE and Papas AS,^8^ the total sugar consumption score was calculated. The sweets were classified into liquids, solid and sticky and slowly dissolving ones. For each time, a sweet was eaten, either at the end of a meal or between meals (at least 20 minutes apart), a frequency chart was drawn. In each group, the frequency of consumption of that group of sweet was multiplied by following numbers: Liquids—5, solid and sticky—10 and slowly dissolving—15. All points were added up for the total score.

Mean, standard deviation and percentage for the respective variable were calculated.

## RESULTS

The final study sample consisted of 35 children (28 males and 7 females).

It was observed that under parental supervision, the children were found to use a variety of tooth brushing aids like: Toothbrush, powered toothbrush and finger; and various agents, like toothpaste, tooth powder and lime and salt mixture. Some were also reported rinsing the mouth using homemade mouthwashes containing indigenous materials. Observations from Table 1 indicate that the one time frequency of brushing in autistic children was seen in maximum number of children (n = 23), while it was found to be declining as it increased to twice (n = 8). None of them was found not to be brushing at any day.

**Table 1:** **Table 1: **Frequency of tooth brushing in autistic children

*Tooth brushing*		*Number of children*	
Twice a day		8	
Once a day		23	
Irregular		4	
Never		0	
Total		35	

Table 2 shows that only 8 children had sugar exposure at meals, while maximum children (n=26) reported sugar exposures in between meals. The total score of sugar consumption per day ranged from a score of 25 to 54.

**Table 2:** **Table 2: **Frequency of sugar consumption by autistic children

*Sugar exposure and score*		*Number of children*	
At meal sugar exposure			
• None		27	
• **≥ **once a day		08	
Between meal sugar exposure			
• None		00	
• **≤ **thrice a day		09	
• > thrice a day		26	
Total sugar exposure			
• None		00	
• **≥ **thrice a day		09	
• > thrice a day		26	
Total score of sugar consumption/day			
• <25		08	
• 25-34		09	
• 35-44		10	
• 45-54		08	

It was seen that only three children were caries free and maximum (n=24) had DEFT greater than 3. The DMFT ranged from 0 to 6.

Table 3 shows the dental treatment needs of the children. The table depicts that 65 to 80% children need preventive care like fluorides and sealants; 40 to 70% need restorative care, while only 23% need surgical treatment, like extraction. The increased number of children requiring crowns can also be attributed to the habit of bruxism seen in these children.

**Table 3:** **Table 3: **Treatment needs

*Treatment needs*		*No. of children*		*Percentage of treatment needs*	
No treatment		0		0	
Fluoride application		27		77.1	
Pit and fissure sealants		23		65.7	
Cavity fillings/pulp care		24		68.6	
Crowns for any reason		15		42.9	
Extraction		8		22.9	
Orthodontic treatment		11		31.4	

Table 4 shows the oral hygiene of autistic children by simplified oral hygiene index for ages 4 to 6 years and 7 to 10 years (deciduous and mixed dentition).^10^ Overall, the OHIS was poor among 14 children.

**Table 4:** **Table 4: **Oral hygiene of autistic children by simplified oral hygiene index for ages 4 to 6 years and 7 to 10 years (deciduous and mixed dentition)

*Number of children*		*OHIS*	
07		Good	
14		Fair	
14		Poor	

## DISCUSSION

The autistic children in the institution spend their time from 9 am to 3 pm performing various activities under the guidance of their caretakers. They have a planned daily schedule which aims at facilitating the child’s independence in activities of daily living and self-care and also a planned afternoon meal is provided in the institution which is devoid of sweets. The institute also arranges for regular diet counseling to the parents.

It has been observed that children under their parental supervision perform active plaque control procedures. According to Nizel AE and Papas AS,^8^ a score of more than 15 is referred to as ‘watch out’ zone which indicates that anything above this is at a greater risk of developing caries. In our study, all the children seem to be in the ‘watch out’ zone despite the best diet counseling and restrictions at the institution. The results of the study indicate that although the ‘at meal’ sugar exposure was close to nil among these institutionalized children, they were seen to be falling in the category of ‘watch out’ zone. The ‘between meal’ sugar exposure is more than three times per day among maximum children inspite of the diet counseling provided to the parents by the institution. This may be attributed to atypical eating behavior and also due to craving for sweets seen in these children.^6,7^

During the clinical examination, the other auxiliary findings seen worth mentioning are the presence of fractured anteriors which may be due to the hyperactive behavior of autistic children leading to trauma; and bruxism which can be attributed to the increased incidence of sleep problems in autistic children.^11^

## CONCLUSION

Autism is one of the five pervasive developmental disorders which are characterized by widespread abnormalities of social interactions and communication, and severely restricted interests and highly repetitive behavior.^12^Although there is currently no cure for autism, early diagnosis and intervention can significantly enhance the child’s social functioning later in life.

After analyzing the present data, a comprehensive oral health plan has been formulated:

- Health education program for parents and teachers together to make them aware of the importance of maintaining good oral care.
- Hands-on demonstration by caregivers in institution to show children brushing teeth with horizontal scrub toothbrushing method using visual pedagogy and also teaching them mouthrinsing habit after every meal.
- Preventive care, like oral prophylaxis, fluorides and sealants, for all the children.
- Use of mouthguard in children (who can tolerate) to protect them from bruxism and self-injurious behavior.

Early detection and early intensive remedial education and behavioral therapy are the most important measures which need to be taken. Patience and time are vital while working with these children.

              ‘The test of morality of a society is what it does for its children’

                                                                                             *Dietrich Bonhoeffer*
